# Preliminary Report of Intravenous Tolvaptan Sodium Phosphate (Samtas^®^) Treatment in Decompensated Heart Failure

**DOI:** 10.3390/jcm13030720

**Published:** 2024-01-26

**Authors:** Makiko Nakamura, Teruhiko Imamura, Koichiro Kinugawa

**Affiliations:** Second Department of Internal Medicine, University of Toyama, 2630 Sugitani, Toyama 930-0194, Japan; nakamura@med.u-toyama.ac.jp (M.N.); kinugawa-tky@umin.ac.jp (K.K.)

**Keywords:** diuretics, hyponatremia, heart failure, responder, vasopressin

## Abstract

**Background:** Tolvaptan sodium phosphate (Samtas^®^; Otsuka Pharmaceutical, Tokyo, Japan) is a novel intravenous aquaretic diuretic aimed at individuals experiencing advanced congestion refractory to conventional diuretics and having difficulty with oral intake. Despite its potential, the true efficacy and practicality of this compound within real-world clinical settings remain obscure. **Methods:** A retrospective analysis of clinical data was conducted, examining trends among consecutive in-hospital patients diagnosed with congestive heart failure who underwent treatment with tolvaptan sodium phosphate at a prominent academic medical center between June 2022 and June 2023. **Results:** Twenty-one patients were enrolled (median age: 75 years, serum N-terminal pro B-type natriuretic peptide: 8941 pg/mL). Among them, 14 patients (67%) received non-invasive/invasive positive-pressure ventilation, and 17 patients (81%) concurrently received intravenous inotropes. Subsequent to the initiation of tolvaptan sodium phosphate treatment, a significant increase in urine volume was observed on the following day (*p* = 0.036). Urine osmolality decreased from 356 (318, 443) at baseline to 247 (176, 333) mOsm/kg after 4 h (*p* = 0.002). No occurrences of hypernatremia were recorded during the therapeutic period. Notably, two patients transitioned from tolvaptan sodium phosphate treatment to continuous hemodiafiltration due to insufficient efficacy. **Conclusions:** In routine clinical practice, intravenous tolvaptan sodium phosphate exhibits potential efficacy and practicability in the majority of congestive heart failure patients exhibiting refractory congestion, unstable hemodynamics, and challenges with oral intake.

## 1. Introduction

Heart failure (HF) poses a substantial global burden, with a noteworthy surge in de novo HF incidence among the elderly population [1,2,3]. Loop diuretics stand as a fundamental component in congestive HF management [4,5]. However, the progressive escalation of loop diuretic dosages stimulates the renin–angiotensin–aldosterone system, thereby causing intravascular hypovolemia, inducing a decline in renal function, and prompting electrolyte imbalances [6,7,8].

Tolvaptan, characterized as an arginine vasopressin type 2 receptor antagonist, is an oral aquaretic agent indicated for managing congestion resistant to conventional diuretics [9,10,11]. Notably, the clinical efficacy of tolvaptan has been documented, particularly among patients concurrently afflicted with chronic kidney disease. This population, often resistant to loop diuretics, experiences benefits such as reduced reliance on loop diuretics, preserved renal function, increased urine output, and alleviated refractory congestion [12,13,14].

However, amidst these advantages, tolvaptan presents several unresolved issues, including drug-induced thirst and hypernatremia [15]. Consequently, its use is contraindicated in individuals facing challenges with oral intake, such as those requiring ventilatory support for decompensated HF. Such patients may not appropriately manage their water intake based on thirst cues, leading to iatrogenic hypernatremia. Additionally, a subset of patients, termed non-responders, fail to exhibit increased urine output following tolvaptan administration due to impaired renal collecting duct function, thereby limiting the drug’s efficacy [16]. Consequently, the applicability of oral tolvaptan may be restricted, particularly in high-acuity settings.

Tolvaptan sodium phosphate (TSP) (Samtas^®^; Otsuka Pharmaceutical, Tokyo, Japan), a prodrug of tolvaptan designed for intravenous administration, has been commercially available in Japan since May 2022 [17,18]. The feasibility of this innovative drug in carefully selected congestive HF patients encountering challenges with oral intake was demonstrated in the TRITON-HF trial [19]. Nevertheless, its viability in real-world clinical practice, encompassing patients with advanced HF and multiple comorbidities, remains uncertain. Herein, we present initial experiences with TSP therapy and explore optimal patient selection and pragmatic management strategies for this novel drug in routine clinical practice.

## 2. Methods

### 2.1. Patient Selection

This retrospective observational study encompassed consecutive patients administered TSP between June 2022 and June 2023 at our institution. Adherence to ethical standards outlined in the “Declaration of Helsinki” was maintained throughout this study, receiving prior approval from the local institutional review board (IRB number R2015154, 11 April 2016). Given the retrospective nature of this investigation and to streamline the process, written informed consent was not required, and an opt-out provision was instituted.

### 2.2. TSP Therapy

Patients experiencing decompensated HF with evident systemic or pulmonary congestion despite receiving intravenous furosemide at doses of 20 mg or higher, and encountering challenges with oral intake or facing restrictions on oral intake, were considered eligible for intravenous TSP administration. The decision for TSP initiation during the index hospitalization was made at the discretion of attending cardiologists.

All patients received monitoring of continuous arterial blood pressure or frequent intermittent non-invasive blood pressure as well as continuous percutaneous oxygen saturation in the cardiac care unit. The initial TSP dosage, either 8 mg or 16 mg daily, was determined by the attending cardiologists. Following TSP commencement, close monitoring of systemic and pulmonary congestion, vital signs, urine output, HF symptoms, and laboratory parameters was conducted to assess TSP efficacy and identify potential drug-related adverse events.

In instances where diuresis post-administration of 8 mg TSP proved insufficient in alleviating congestive symptoms, the dosage was escalated to 16 mg. Upon stabilization of patients’ hemodynamics and their ability to orally ingest medications, a transition from intravenous TSP to oral tolvaptan was considered. Termination of TSP was considered upon sufficient relief from congestion or in cases where hypovolemia or any other drug-related adverse events surfaced.

### 2.3. Collected Data

The observational duration primarily corresponded to the period of TSP administration. If patients underwent TSP treatment for a duration exceeding 10 days, the observational period was standardized to 10 days.

Baseline characteristics encompassed underlying heart disease and comorbidities, along with pertinent laboratory parameters such as plasma B-type natriuretic peptide, serum N-terminal pro B-type natriuretic peptide (NT-proBNP), estimated glomerular filtration rate (eGFR), echocardiographic data, and concomitant medications.

Data were also collected at the conclusion of the observational period. Specifically, urine osmolality measurements were obtained at baseline, 2 h post-administration, 4 h post-administration, and the subsequent day’s baseline.

### 2.4. Endpoints

#### 2.4.1. Safety Analyses

The primary endpoint focused on drug-related adverse events necessitating the discontinuation of the drug or unexpected interventions throughout the observational period. Secondary endpoints comprised occurrences of hypernatremia, hypokalemia, and renal impairment within the observational period. Worsening renal function was defined as a serum creatinine increase >0.3 mg/dL [20].

#### 2.4.2. Efficacy Analyses

The primary endpoint aimed to assess the augmentation in urine volume over the 24 h TSP therapy duration. Secondary endpoints included a decline in urine osmolality during the 24 h TSP therapy and a reduction in serum NT-proBNP levels throughout the observational period.

### 2.5. Statistical Assessments

Statistics were conducted using JMP pro ver17.0 (SAS Institute Japan. Tokyo, Japan). Variables attaining a significance level of *p* < 0.05 were deemed statistically significant. Continuous data are expressed as median values along with interquartile ranges, irrespective of their distribution normality, owing to the small sample size. Categorical data are presented as numbers and percentages. Comparison between baseline and post-TSP therapy continuous data was executed using the Wilcoxon signed-rank test.

## 3. Results

### 3.1. Baseline Characteristics

A total of 21 patients were included (Table 1). All patients had congestive heart failure refractory to conventional diuretics and difficulty with oral intake—an indication of TSP. Median age was 75 (68, 81) years old and 7 (33%) were men. Median systolic blood pressure was 99 (91, 105) mmHg and median eGFR was 35.8 (21.5, 50.9) mL/min/1.72 m^2^. All patients received loop diuretics at a furosemide-equivalent dose of 20 (20, 40) mg/day.

Fourteen patients (67%) received intravenous dobutamine infusion. Five patients (24%) received carperitide infusion. Fourteen patients (67%) had received non-invasive/invasive positive-pressure ventilation support.

### 3.2. Trajectory of TSP Therapy

TSP was administered for 5 (3, 8) days. Among the patient cohort, 12 individuals underwent a transition from TSP to oral tolvaptan. Conversely, in nine patients, cessation of TSP occurred, with seven patients exhibiting an improvement in congestion while two patients experienced a deterioration in their clinical condition, prompting TSP termination.

### 3.3. Safety Analysis

TSP was introduced at an initial dosage of 8 mg/day for 14 patients, with 6 of these individuals requiring up-titration to 16 mg/day. Additionally, seven patients commenced TSP therapy at a starting dose of 16 mg/day, while one among them had their dose elevated to 21 mg/day.

Remarkably, no drug-related adverse events necessitating TSP termination occurred during the therapeutic period. This included the absence of severe hypotension, hypovolemia, critical thirst, and hypernatremia, except for two patients who commenced continuous hemodiafiltration to manage systemic congestion.

Patient A experienced exacerbation of heart failure concomitant with a urinary tract infection leading to septic shock. Although TSP elicited a slight elevation in urine volume from 250 mL/day to 350 mL/day, this increment proved insufficient in ameliorating systemic edema, ultimately prompting termination of TSP therapy on day 6. Despite the initiation of renal replacement therapy, regrettably, the patient’s condition deteriorated, culminating in their passing on day 11.

Another patient (Patient B), afflicted with severe cardiac systolic dysfunction compounded by pneumonia, concluded TSP therapy on day 6. Similar to the previous case, this patient exhibited only a marginal increase in urine volume subsequent to the commencement of TSP. Notably, her baseline urine osmolality measured at 191 mOsm/kg remained largely unchanged at 193 mOsm/kg four hours later. Although continuous hemodiafiltration was initiated on day 9, unfortunately, the patient passed away on day 22.

There were three other patients (14%) who had worsening renal function during the observational period. Two of three had very depressed left ventricular ejection fractions and received two and three types of intravenous inotropes, respectively. The remaining one patient was elderly, frail, and anorexic with a body mass index of 14.4 and preserved left ventricular ejection fraction; they had not received vasodilators nor vasoactive agents.

**Figure 1:**
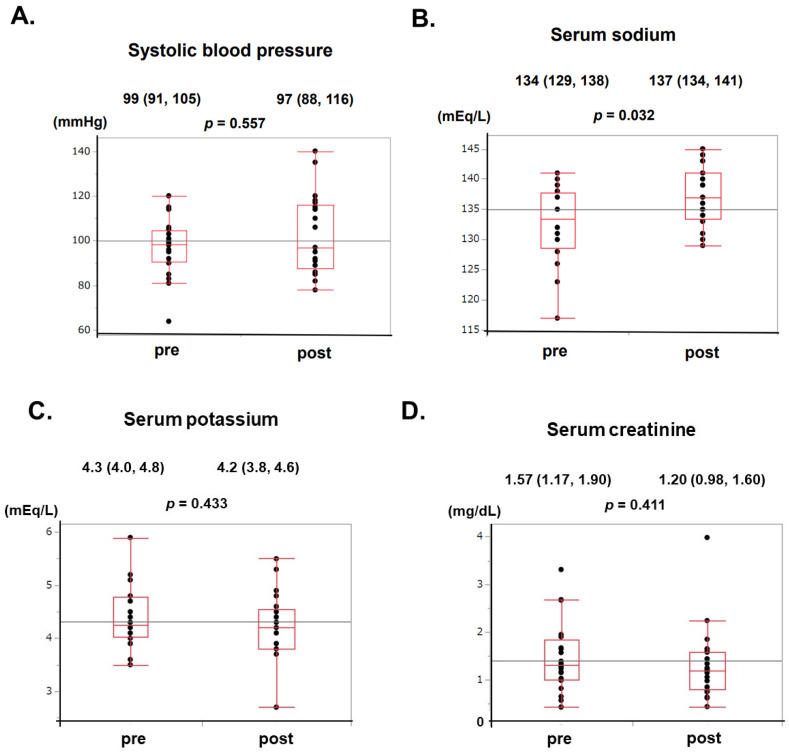
Comparison of systolic blood pressure (**A**), serum sodium (**B**), serum potassium (**C**), and serum creatinine (**D**) levels at baseline and after administration of intravenous TSP.

As a whole, systolic blood pressure remained unchanged from 99 (91, 105) mmHg to 97 (88, 116) mmHg (*p* = 0.557; Figure 1A), whereas serum sodium levels increased significantly from 134 (129, 138) mmHg to 137 (134, 141) mmHg (*p* = 0.032; Figure 1B). No patients had hypernatremia. Serum potassium and creatinine levels remained unchanged from 4.3 (4.0, 4.8) to 4.2 (3.8, 4.6) mEq/L (*p* = 0.433; Figure 1C) and from 1.31 (1.00, 1.84) to 1.20 (0.81, 1.59) mg/dL, respectively (*p* = 0.411; Figure 1D).

### 3.4. Efficacy Analysis

**Figure 2:**
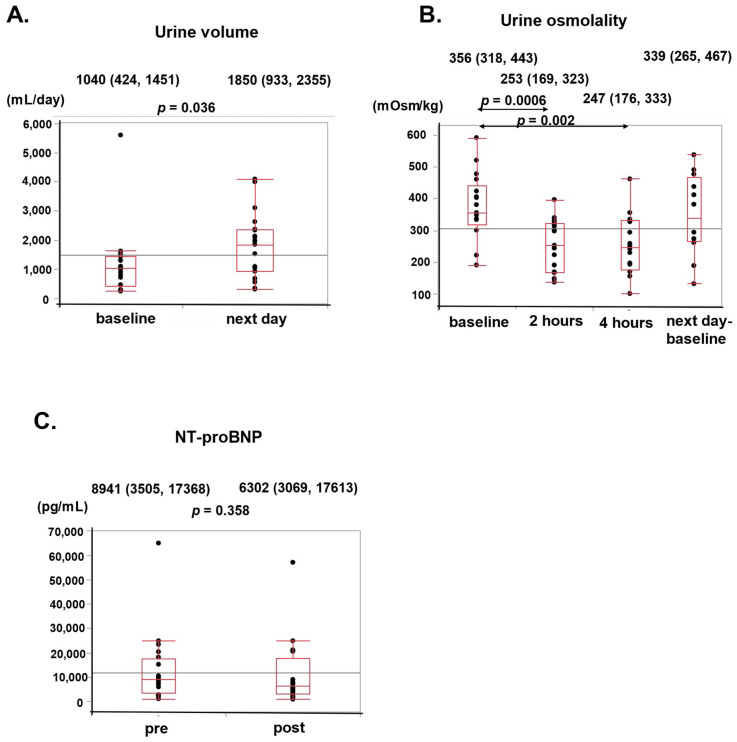
Comparison of urine volume (**A**) and serum NT-pro BNP (**C**) at baseline and after administration of intravenous TPS. Comparison of urine osmolality (**B**) at baseline, at 2 h later, at 4 h later, and at the next day’s baseline after administration of intravenous TSP. NT-proBNP, N-terminal pro-B-type natriuretic peptide.

As a primary outcome, urine volume increased significantly from 1040 (424, 1451) to 1850 (933, 2355) mL/day the next day (*p* = 0.036; Figure 2A).

As a secondary outcome, urine osmolality significantly decreased from 356 (318, 443) mOsm/kg at baseline down to 253 (169, 323) mOsm/kg at 2 h, and 247 (176, 333) mOsm/kg at 4 h after TSP administration (*p* < 0.001 and *p* = 0.002, respectively; Figure 2B). Urine osmolality increased again up to 339 (265, 467) mOsm/kg the next day. As another secondary outcome, serum NT-pro BNP tended to decrease from 8641 (3505, 17368) pg/mL to 6302 (3069, 17613) pg/mL (*p* = 0.358; Figure 2C).

## 4. Discussion

In this investigation, we aimed to evaluate the safety and effectiveness of TSP among patients experiencing decompensated heart failure resistant to conventional diuretics and encountering challenges with oral intake in a real-world clinical setting. Our preliminary data involved the administration of intravenous TSP to twenty-one patients.

During the course of intravenous TSP therapy, minimal occurrences of drug-related adverse events emerged, such as severe hypotension, critical thirst, and hypernatremia necessitating the cessation of the drug. Notably, these events transpired despite the advanced stage of heart failure in these individuals, who faced difficulties managing water intake independently. However, two patients subsequently required continuous hemodiafiltration.

Urine volume displayed an increase subsequent to TSP administration, while urine osmolality exhibited a decrease either 2 or 4 h post-administration. These observations reflect the impact of TSP on urinary parameters within this cohort of patients.

### 4.1. Safety Analyses

Despite our cohort being in an advanced stage of heart failure and experiencing difficulties in managing water intake independently, notably, there were scarce critical drug-related adverse events necessitating the termination of TSP. Instances of severe hypotension or electrolyte imbalances inducing new-onset arrhythmias were notably absent within our patient group.

The incidence of worsening renal function was 14%, which was lower compared to that in previous reports regarding conventional diuretics [20,21]. There are similar previous reports regarding the reno-protective effect of oral tolvaptan [7].

It is noteworthy that none of the patients developed hypernatremia, which presents a notable advantage over conventional oral tolvaptan, particularly in patients facing challenges with oral intake. The precise mechanism behind this observation remains uncertain; however, one potential explanation could be attributed to the relatively shorter half-life of TSP in the bloodstream, which may contribute to this enhanced safety profile [17].

However, it is imperative to emphasize the necessity of diligent monitoring encompassing vital signs, heart failure symptoms, electrocardiogram readings, and urine parameters throughout the course of TSP therapy. Such meticulous observation plays a pivotal role in preventing potential drug-related adverse events.

### 4.2. Efficacy Analysis

Urine volume increased significantly following the initiation of TSP accompanying the immediate decrease of urine osmolality. The time to peak plasma concentration of 16 mg TSP was around 1.5 h in a phase II trial [19]. Urine volume increased to a maximum level at 1–2 h following intravenous TSP administration in a phase III trial [19]. We were able to validate that, in our cohort, characterized by more advanced heart failure and multiple comorbidities for the representation of real-world patients, urine osmolality notably reached its lowest levels around 2 to 4 h post-TSP administration. This swift and pronounced response, reflective of a real-world clinical population, holds significant practical implications, particularly in patients experiencing acute heart failure with unstable hemodynamics necessitating an immediate therapeutic intervention.

However, it is crucial to note that two patients demonstrated a lack of clinical response to TSP. These individuals were afflicted by septic or cardiogenic shock and presented with impaired renal function. Similar to observations with oral tolvaptan, the residual function of the kidney collecting duct might play a pivotal role in determining the responsiveness to tolvaptan therapies [22]. Optimal patient selection and therapeutic strategies for non-responders to TSP remain the next concerns.

### 4.3. Clinical Implication

Tolvaptan, owing to its robust aquaresis, carries a risk of hypernatremia and hypovolemia. It is contraindicated in patients facing challenges with oral intake or lacking a sense of thirst. However, our observation with TSP revealed a safe administration profile devoid of these adverse events, even among patients unable to self-regulate water intake. This finding holds promising implications, particularly considering that a majority of individuals with advanced heart failure rely on respiratory support or are restricted from water intake. These patients stand as potential candidates for TSP therapy.

Nonetheless, it is crucial to underscore the necessity for vigilant monitoring of vital signs, hemodynamics, and laboratory parameters throughout TSP therapy to prevent the occurrence of such adverse events.

The prompt decline in urine osmolality observed in the majority of candidates in our study serves as a direct indicator of the response to TSP, signifying an increase in free water excretion through urine. We strongly advocate for the regular measurement of urine osmolality, both at baseline and at subsequent time intervals, as this practice enables the anticipation of response to TSP.

One distinct advantage of TSP over conventional oral tolvaptan lies in the ability to promptly adjust the dosage of continuously and intravenously administered TSP based on the response observed. For instance, when urine volume fails to increase despite the preservation of baseline urine osmolality, indicating a positive response, it is advisable to consider up-titrating the TSP dosage for these responders. This tailored approach allows for timely and personalized adjustments in TSP dosage to optimize therapeutic outcomes.

### 4.4. Study Limitations

Our study was limited by the small sample size within a single institution, attributed to the recent availability of TSP since May 2022. There was no control arm and data regarding the transition of congestion symptoms as well as duration of hospitalization were missing. The dose of loop diuretics was low as 20 (20, 40) mg daily—in this study, which is similar to that in recent registry data in Japan [3]. Dosages of loop diuretics in Japan are fairly lower than those currently recommended in international guidelines, and the use of tolvaptan during hospitalization for acute decompensated heart failure patients who are resistant to other diuretics including loop diuretics is recommended at a class IIa level [9]. In this study, the use of intravenous dobutamine was relatively high at 67%. In Japan, dobutamine is a first-line inotrope with a class IIa recommendation [9] and widely used in patients with cardiogenic shock and not only reduced ejection fraction but also in patients with valvular heart disease, right heart dysfunction, and diastolic dysfunction. This study serves as a proof-of-concept, and future larger-scale studies are imperative to validate and substantiate our preliminary findings. Nonetheless, we consider this study to be a valuable initial exploration that showcases the feasibility and effectiveness of TSP in real-world clinical scenarios encompassing individuals with advanced heart failure unresponsive to conventional diuretics and presenting with multiple comorbidities.

Additionally, our study lacked a control group. Ethical considerations and the current standards of care make it challenging and ethically inappropriate to manage individuals with congestive heart failure resistant to conventional diuretics without utilizing TSP in the present healthcare landscape. This limitation underscores the difficulty in establishing a control arm for comparison in this context.

We acknowledge the potential influence of other medications on clinical outcomes in our study cohort. However, it remains challenging from a clinical standpoint to manage such severe patients without adhering to guideline-directed medical therapy. The complexity of their condition often necessitates the use of multiple medications to address various aspects of their health, making it difficult to isolate the precise impact of individual drugs on observed outcome.

## 5. Conclusions

The administration of intravenous TSP appears to offer feasibility and efficacy among patients grappling with decompensated heart failure resistant to conventional diuretics, particularly those encountering challenges in self-regulating water intake. However, our study serves as a preliminary proof-of-concept due to the recent clinical introduction of TSP. Larger-scale studies are imperative to substantiate and validate our findings, and should aim to establish optimal patient selection criteria for TSP utilization and devise robust therapeutic strategies supported by comprehensive evidence.

## Figures and Tables

**Table 1:** Baseline characteristics.

	Total Patients (*n* = 21)
Demographics	
Age (years)	75 (68, 81)
Male sex	7 (33%)
Body mass index	24.3 (19.6, 24.8)
Valvular disease	7 (33%)
Hypertrophic cardiomyopathy	1 (5%)
Dilated cardiomyopathy	2 (10%)
Ischemic etiology	2 (10%)
Pericarditis	1 (5%)
Myocarditis	1 (5%)
Infectious endocarditis	1 (5%)
Pulmonary artery hypertension	1 (5%)
Aortic dissection	1 (5%)
Atrial fibrillation	14 (67%)
Systolic blood pressure (mmHg)	99 (91, 105)
Diastolic blood pressure (mmHg)	64 (57, 76)
Heart rate (bpm)	89 (77, 100)
Laboratory data	
White blood cell (×10^2^/μL)	7.6 (5.6, 10.4)
Hemoglobin (g/dL)	10.1 (9.1, 11.6)
Platelet (×10^3^/μL)	15.4 (10.2, 22.4)
Albumin (mg/dL)	2.8 (2.5, 3.2)
Total bilirubin (mg/dL)	1.3 (0.7, 1.8)
Blood urea nitrogen (mg/dL)	29.8 (20.9, 47.3)
Serum creatinine (mg/dL)	1.31 (1.00, 1.84)
Estimated glomerular filtration rate (mL/min/1.73 m^2^)	35.8 (21.5, 50.9)
Serum sodium (mEq/L)	134 (129, 138)
Serum potassium (mEq/L)	4.3 (4.0, 4.8)
C-reactive protein (mg/dL)	3.31 (1.39, 7.42)
Plasma B-type natriuretic peptide (pg/mL)	496 (244, 904)
Serum N-terminal pro B-type natriuretic peptide (pg/mL)	8941 (3504, 17368)
Urine osmolality (mOsm/kg)	356 (318, 443)
Echocardiographic data	
Left ventricular ejection fraction (%)	52 (27, 64)
Left ventricular ejection fraction <40%	8 (38%)
Aortic stenosis (≥moderate)	3 (14%)
Aortic regurgitant (≥moderate)	0 (0%)
Mitral valve stenosis (≥moderate)	2 (10%)
Mitral valve regurgitant (≥moderate)	6 (29%)
Tricuspid valve stenosis (≥moderate)	0 (0%)
Tricuspid valve regurgitant (≥moderate)	7 (33%)
Medication	
ACEI/ARB	6 (29%)
ARNI	4 (19%)
Beta blocker	10 (48%)
Mineralocorticoid receptor antagonist	9 (43%)
SGLT2 inhibitor	2 (10%)
Loop diuretics	21 (100%)
Dose of loop diuretics (furosemide equivalent; mg)	20 (20, 40)
Previous oral tolvaptan medication	8 (38%)
Dobutamine	14 (67%)
Milrinone	5 (24%)
Carperitide	5 (24%)
Nitroglycerin	1 (4.7%)
Noradrenaline	5 (24%)
Dopamine	5 (24%)
Non-invasive/invasive positive-pressure ventilation	14 (67%)

ACE; angiotensin-converting enzyme inhibitor, ARB; angiotensin II receptor blocker, ARNI; angiotensin receptor–neprilysin inhibitor, SGLT2; sodium–glucose cotrasporter-2.

## Data Availability

Data are available from the corresponding author upon reasonable request.
